# Progress in Cancer

**Published:** 1903-08-29

**Authors:** 


### Progress in Cancer.

A comparative study of cancer mortality, as revealed by the statistics available from various countries of the world, has been made by Dr. Alfred Wolff.[1] The countries which furnished statistics were England and Wales, Scotland, Ireland, France, Germany, Austria-Hungary, United States, Switzerland, Italy, Holland, Scandinavia, other countries (including India, Australia, etc.). The general conclusions are rather more negative than positive, and it is disappointing that more cannot be obtained from such a wide field. But the writer has started to prove no preconceived theory, and the conclusions should thus be more valuable on that account, being as they are the result of a bird's eye view. It seems that no definite attraction for any geological stratum is shown, for that "strata of every geological period show alike areas of high and others of low mortality, although the former are perhaps more often found on recent deposits." It seems very doubtful whether there is any persistent prevalence of cancer among populations living in river valleys, and whether, where this does occur, it may not be due to some superadded condition. In nearly every district where cancer mortality is high there is forest land, or at least it is well wooded. This is noticeable in France and Germany especially, and also in Austria, Switzerland and Italy. Yet areas which have been deforested have a low mortality. It cannot be doubted, he holds, that trees have a very serious and potent influence on those who live in their neighbourhood; for they seem, beyond question, to make men prone to suffer from cancer. With regard to food, there is no evidence that the consumption of large quantities of meat is a cause. Cancer is not scarce among races who eat very little or no meat at all, *e.g.*, Hindus and Chinese, Italians and Norwegians. Fish-eaters may perhaps have a somewhat increased tendency to malignant disease; but yet the Bretons, who are largely a fishing population, suffer but little.

A study of these statistics points most forcibly to a connection between the consumption of beer and cider as beverages and the incidence of cancer. Especially is this to be noticed in (*a*) Bavaria, where a larger quantity of beer, per head, is drunk than in any other country, and the cancer rate is extremely high, and in (*b*) Lille, where far more beer is drunk than in any other French town, and which gives the largest percentage of cancer for persons over 40 years of age. Alcoholism *per se* does not seem to bear a close connection with the cancer rate. The latter is high in many classes of individuals which are intemperate, but this may be due to associated causes. With regard to occupation, besides the well-known tendency for chimney-sweeps to cancer, which is far above the average, it is also so in the liquor trade. The coal miners in Derby and Nottingham show twice the death-rate that those in Monmouthshire and South Wales do. Seamen, blacksmiths, and masons show an exceedingly high rate in the United States, and among females there is a very high cancer rate among nurses and domestic servants: the rate between 45 and 64 being double, and above 65 treble the average.

There is some evidence that Teutonic and Scandinavian races are more liable than the Latin, Sclav, and Celtic races. In the United States it is found that change of environment has in many cases materially altered the comparative liability to suffer from malignant growth. This fact suggests the probability that it is not race alone, but the diet and habits of different peoples which contribute the important element of the difference. He holds that the existence everywhere of areas of high and low mortality from cancer is difficult of explanation by any theory of the etiology of the disease which excludes the existence of a specific cause. Many observers testify to the existence of infected houses. The persistent-irritation theory may be harmonised with the bacillus theory. It may be that, like the B. lepræ, it remains dormant, even in favourable soil, until urged into activity by an exciting cause in the form of some chronic local irritation. It is possible that different soils are associated with high cancer mortality only in so far as they encourage growths of certain forms of vegetation.

The definite conclusions of a positive character which seem to result from the present inquiry may be summed up in the following :—1. That certain races are especially prone to cancer, more particularly the Scandinavian and different branches of the Germanic family. 2. That cancer is more prevalent in districts where beer is the staple drink, and the excess is in some degree proportionate to the amount consumed per head. 3. That cancer tends to cause an excessive mortality in regions abounding in water, but to a much more marked extent when these are covered with woods and forests. The mortality is also usually high along the valleys of rivers flowing from such districts. 4. That the regional distribution of cancer indicates the probability that the disease is due to a specific cause.

From the statistics of Dundee, Templeman [2] considers the questions : 1. Has the mortality from cancer increased ? 2. Is the increase real or apparent ? 3. If real, how is it to be accounted for ? His conclusions from comparison and examination of these statistics are that—1. In the period under examination the death-rate from cancer has more than doubled per 10,000 over 20 years. 2. This increase is greatest at ages over 45—is common to both sexes, but more marked in the male sex, though the actual mortality is greatest among females. 3. In females it is chiefly due to an increase in malignant affections of the abdominal viscera. 4. Uterine cancer and cancer of the breast in females have increased though not in any marked degree. 5. Cancer of the rectum also shows a slight increase in both sexes. 6. In males the highest mortality is from cancer of the abdominal viscera. 7. In males cancer of the mouth and upper digestive tract has also greatly increased. 8. Cancer of parts to be regarded as accessible has increased, as well as that of parts which are not so accessible, and where diagnosis is more difficult; but the increase of the latter is out of all proportion to that in the former classes. 9. Improvement in clinical and pathological diagnosis has helped to swell the returns of death from cancer.

Speaking concerning cancer of the tongue and the advisability of early operation, Butlin [3] says "there is perhaps no part of the body in which early operation for cancer is called for so urgently as in the tongue ; and there is no part of the body in which early operation is likely to be attended by better results." The diagnosis is most easily made, and it frequently developes under one's eye from some recognised precancerous condition or predisposing cause. In his own statistics he has been successful in 28 out of 113 (25 per cent.) cases of cancer of the tongue. One case was operated on 19 years previously, a large part of the tongue being removed, with glands, and has had no further trouble in the tongue or neck. Another is well after three years, although there was an extensive dissection of the anterior triangle of the neck, with removal of the tongue and the floor of the mouth. Both of these cases were verified microscopically. Guinard [4] had a case of recurrent lingual cancer in a man of 69, who had been free of the disease for 18 years after operation, and he warns us that had this patient been taking any drugs or undergoing serum injections, undue credit would have been given to this. Butlin has obtained excellent results in his cases, but there are 97 unsuccessful cases, of which 14 died of the operation ; 12 of some other disease than cancer ; 29 with recurrence in the mouth were absolutely unsuccesful ; one had secondary disease ; 41 had secondary disease in the glands, not in the mouth. Reviewing the want of success in this list he urges that much more attention should be given to the predisposing causes, such as chronic superficial glossitis and leucoplakia, or those conditions which being so frequently precursors of malignant disease, are termed precancerous conditions. The most common of these is a warty growth on the tongue or a chronic ulcer from a tooth. An area of leucoplakia which getting thicker and more prominent may soften in the centre is very indicative of ensuing cancer ; while lumps and nodules and old chronic ulcerations may only rarely become cancerous. The former should be excised early, and the reason why this is not done more often is frequently a fear, too often shared by medical men of not being able to speak plainly, or to eat properly, after removal of part of the tongue. This is absolutely groundless. All warts, warty growths, and the causes of chronic ulcerations should be removed. If an ulcer does not disappear in seven to ten days after the cause is removed, then it should be excised. In bad chronic superficial glossitis, where the tongue is constantly being irritated, and where there is a constant dread of cancer ensuing, he advises removal of that part of the tongue, even if there is no sign of cancer present at the time. If the mucous membrane beneath be healthy, this will form an excellent covering to the stump, and the after effects will be so good that in speaking and eating there will be little difference from the normal.

Further examples of the advantage of early operation are cited by Crawford Benton [5] who has had five early cases of carcinoma of the breast, with the following results :—One case is well 18 years after operation, the second 8 years after. The third operated 25 years ago was well for 15 years when a tumour developed in the other breast, which was removed ; five years later an epithelioma of the vulva necessitated removal. She has since been well. The fourth case was one of a cystic condition with "precancerous cells," the patient being well four years after this breast was removed ; and the fifth was a cystic swelling of breast becoming cancerous the patient being well after $3\frac{1}{2}$ years. The pathological condition was verified microscopically.

[1] Brit. Med. Jour., May 16. [2] Ibid., Feb. 14. [3] Ibid. [4] Ibid., May 23. [5] Ibid., April 11.

(*To be continued.*)

